# NETs: organ-related epigenetic derangements and potential clinical applications

**DOI:** 10.18632/oncotarget.10598

**Published:** 2016-07-13

**Authors:** Mauro Cives, Valeria Simone, Francesca Maria Rizzo, Franco Silvestris

**Affiliations:** ^1^ Department of Biomedical Sciences and Human Oncology, Section of Internal Medicine and Clinical Oncology, University of Bari “Aldo Moro”, Bari, Italy

**Keywords:** carcinoid tumors, DAXX, ATRX, MEN1, DNA methylation

## Abstract

High-throughput next-generation sequencing methods have recently provided a detailed picture of the genetic landscape of neuroendocrine tumors (NETs), revealing recurrent mutations of chromatin-remodeling genes and little-to-no pathogenetic role for oncogenes commonly mutated in cancer. Concurrently, multiple epigenetic modifications have been described across the whole spectrum of NETs, and their putative function as tumorigenic drivers has been envisaged. As result, it is still unclear whether or not NETs are epigenetically-driven, rather than genetically-induced malignancies. Although the NET epigenome profiling has led to the identification of molecularly-distinct tumor subsets, validation studies in larger cohorts of patients are needed to translate the use of NET epitypes in clinical practice. In the precision medicine era, recognition of subpopulations of patients more likely to respond to therapeutic agents is critical, and future studies testing epigenetic biomarkers are therefore awaited. Restoration of the aberrant chromatin remodeling machinery is an attractive approach for future treatment of cancer and in several hematological malignancies a few epigenetic agents have been already approved. Although clinical outcomes of epigenetic therapies in NETs have been disappointing so far, further clinical trials are required to investigate the efficacy of these drugs. In this context, given the immune-stimulating effects of epidrugs, combination therapies with immune checkpoint inhibitors should be tested. In this review, we provide an overview of the epigenetic changes in both hereditary and sporadic NETs of the gastroenteropancreatic and bronchial tract, focusing on their diagnostic, prognostic and therapeutic implications.

## INTRODUCTION

Neuroendocrine tumors (NETs) include a heterogeneous group of malignancies characterized by a relatively indolent rate of growth and a propensity to secrete a variety of hormones and biogenic amines. They arise from neuroendocrine cells, which are mainly located throughout the length of the gastroenteropancreatic (GEP) tract and the bronchopulmonary tree. The majority of NETs are sporadic, but they can also occur in the context of inherited familial syndromes, such as multiple endocrine neoplasia type 1 (MEN1), Von-Hippel Lindau syndrome, tuberous sclerosis and neurofibromatosis type 1, thus suggesting a causal role for genetic alterations during the tumorigenic process [1].

In recent years, a very heterogeneous picture of the genetic landscape of well-differentiated foregut, midgut and hindgut NETs has been depicted. Mutations of covalent histone modifiers including *MEN1*, *PSIP1*, *SETD1B* and members of the Polycomb complex have been observed in 40% of pulmonary carcinoids, and alterations in chromatin-remodeling genes have been described as sufficient to drive early steps in lung NET tumorigenesis [2]. In pancreatic NETs (pNETs), mutations of the epigenetic regulators *MEN1* and *DAXX/ATRX* have been described in 44% and 43% of tumors respectively, while alterations of the mammalian target of rapamycin (mTOR) pathway have been found in 14% of the specimens [3]. Whole-genome and -exome sequencing has demonstrated that small bowel NETs are mutationally quiet, with a mutational burden of 0.1 somatic single nucleotide variants (SSNVs) per 10^5^ nucleotides. Accordingly, recurrent mutations in the cyclin-dependent kinase inhibitor gene *CDKN1B* have been identified in only ~8% of tumors, in the absence of other obvious pathogenetic genomic alterations [4]. However, multiple epigenetic aberrations have been recently demonstrated in small bowel NETs, and their involvement in disease pathogenesis has been postulated [5]. Although patterns of gene mutations are highly diverse in NETs of different primary sites, classical oncogenes or tumor suppressors implicated in the development of many solid tumors (such as *P53*, *RB* or *KRAS*) do not appear to play a major role in the pathogenesis of any NETs [6]. In contrast, epigenetic dysregulation and/or alterations of the chromatin remodeling machinery seem to be a common element across different histologies. Thus, whether low-to-intermediate grade NETs are genetically-driven neoplasms or epigenetically-induced malignancies remains a legitimate though unanswered question.

**Figure 1:**
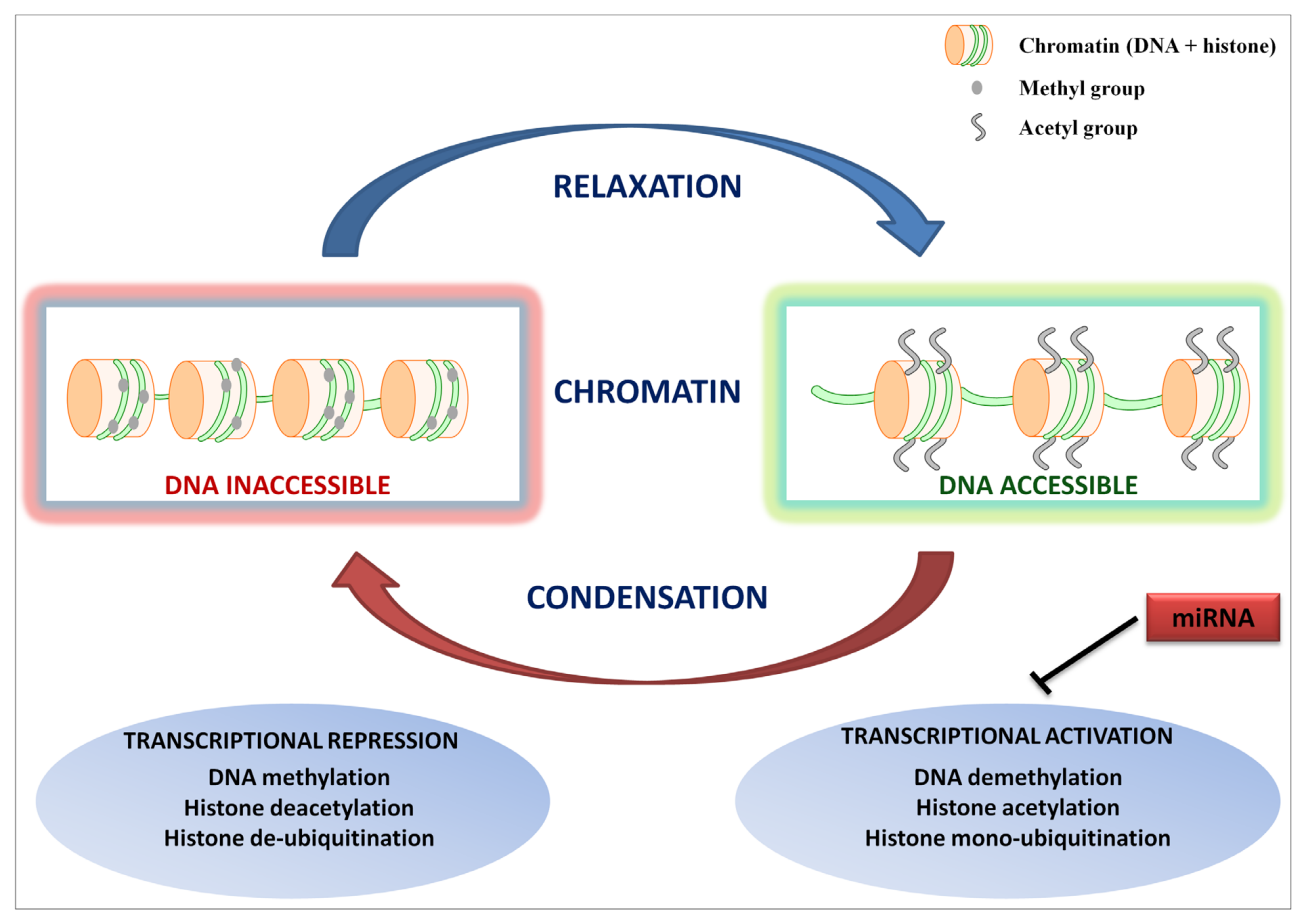
Epigenetic regulation of gene expression Epigenetic alterations such as DNA methylation and/or histone modifications modulate the accessibility of genes to the transcriptional machinery by inducing either a relaxed/open or condensed/closed chromatin configuration. miRNAs concur to regulate the cell phenotype by repressing the expression of gene transcripts.

Epigenetic modifications such as DNA methylation or histone acetylation, methylation and phosphorylation can cause heritable changes in gene expression without concomitant alterations in the genome of a cell. DNA methylation occurs primarily within the CpG islands located in the promoter regions and dictates the transcriptional potential of downstream target genes. Concomitantly, covalent histone modifications determine how DNA is packaged in nucleosomes, thus modulating the accessibility of underlying genes to transcription factors [7]. In addition, microRNAs (miRNAs), small single-stranded RNA molecules of ~19-22 nucleotides, regulate the gene expression at the post-transcriptional level, and have recently emerged as prominent epigenetic regulators [8]. Acting combinatorially, these mechanisms concur to determine the cellular phenotype, and there is increasing evidence that epigenetic aberrations are as relevant as gene mutations in the cancer pathogenesis (Figure 1) [9].

In this review, we provide an overview of the current knowledge on epigenetic changes in both hereditary and sporadic NETs of the GEP and bronchial tract, and focus on both diagnostic, prognostic and therapeutic implications that the NET epigenome profiling carries in the precision medicine era.

## THE EPIGENETIC LANDSCAPE OF NETS

During the past decade, next-generation DNA and RNA sequencing methods have provided a more detailed picture of the epigenetic landscape of NETs. In particular, genome-wide approaches have contributed to define a molecular classification of these malignancies, thus paving the road for new diagnostic tools and innovative personalized therapies. Neuroendocrine lineage allocation is partly caused by progressive accumulation of complex layers of epigenetic modifications during the differentiation process from pluripotent endodermal cells [10, 11]. Although phenotypic stability of the differentiated cell state is secured by the so called “epigenetic memory” (reviewed in [12]), heterogeneous epigenetic profiles have been shown in NETs of different primary sites, thus suggesting underlying difference in the tumorigenic process, microenvironment-driven modulation of epigenetic states, and/or their possible correlation with the biological aggressiveness of these diverse neoplasms.

### Familial neuroendocrine syndromes

Genetic and clinical features of familial neuroendocrine syndromes are summarized in Table 1. MEN1 is an autosomal-dominant syndrome characterized by tumors of the anterior pituitary, parathyroid glands and pancreaticoduodenal neuroendocrine cells, most commonly gastrinomas. It is caused by an inactivating mutation of the *MEN1* gene, which encodes for menin, a nuclear protein implicated in cell division, genome stability, and transcription regulation *via* histone methylation. Up to 10% of patients with MEN1 syndrome may not harbor mutations in the coding regions of the *MEN1* gene, but in the gene promoter or untranslated regions, challenging the genetic diagnosis [13]. As a constituent of a multiple protein complex displaying a histone H3 lysine 4 methyltransferase activity, MEN1 has a critical role in chromatin remodeling. In particular, MEN1 acts as either repressor or activator of gene transcription through interaction with a plethora of histone deacethylases (HDACs) and histone methyltransferases including PRMT5 and SUV39H1. Epigenetic silencing of the Hedgehog pathway, of the homeobox gene *GBX2* as well as of the gastrin-encoding gene *GAST* has been reported downstream of MEN1 [14–16]. On the other hand, transcriptional activation of the HOX cluster (*HOXA9*, *HOXC6*, and *HOXC8*) and cyclin-dependent kinase inhibitor (*CDKN1B*, *CDKN2C*) genes has been documented, but the biologic consequences on pNET tumorigenesis need to be clarified [17–19]. To elucidate the genome-wide transcriptional modifications induced by MEN1 through epigenetic remodeling in pancreatic islets, a recent study integrated gene expression profile analysis and histone H3 lysine 4 trimethylation (H3K4me3) mapping, and identified insulin-like growth factor 2 mRNA binding protein 2 (*IGF2BP2*) gene as a target subjected to MEN1 dynamic regulation. IGF2BP2 interferes with IGF2 translation during the embryonic development, and its dysregulation upon MEN1 loss might play a role in pNET pathogenesis [20].

**Table 1:** Familial neuroendocrine syndromes: genetic and clinical features

Syndrome	Causative gene	Gene location	Protein	GEP-NET type (penetrance)
MEN1	*MEN1*	11q13	Menin	Gastrinoma (40%) Non-functioning pNET (20%) Insulinoma (10%) Glucagonoma <1% VIPoma <1% Gastric carcinoid 10%
VHL syndrome	*VHL*	3p25	VHL	Non-functioning pNET (12-17%)
Tuberous sclerosis	*TSC1*/*TSC2*	9q34/16p13	Hamartin/tuberin	pNET (<5%)
NF1	*NF1*	17q11.2	Neurofibromin	Somatostatinoma (6%)

In neurofibromatosis type 1 and tuberous sclerosis syndrome, epigenetic silencing of the wild-type *NF1* or *TSC2* genes has been proposed as a possible tumorigenic event, in accordance with the Knudson's two-hit hypothesis [21, 22]. Although data in lung and GEP-NETs arising in the context of Von-Hippel Lindau syndrome (VHL) are lacking, there is evidence that mutations of multiple chromatin remodelers including the histone methyltransferase SETD2 and the histone demethylases UTX and JARID1C may contribute to the progression of VHL-associated clear renal cell carcinoma [23].

### Pancreatic NETs

**Figure 2:**
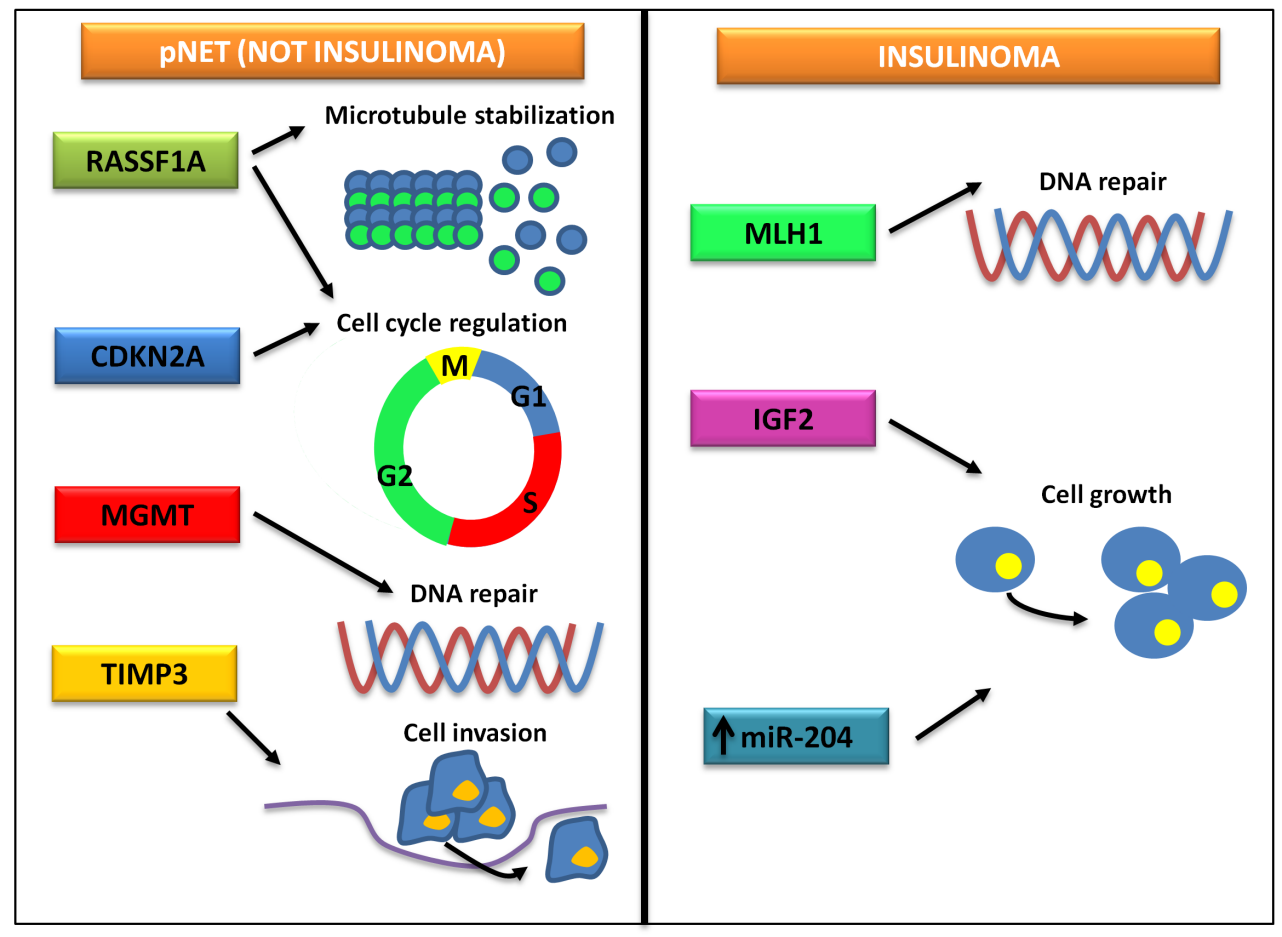
Frequent epigenetic modifications in insulinomas and other pNETs While the epigenetic landscape of insulinomas is characterized by alterations of the signaling of MLH1 and IGF2, non-insulinoma pNETs are defined by a different pattern of epigenetic changes, eventually leading to cell cycle dysregulation, increased cell motility and chemoresistance.

A number of studies have investigated the epigenetic changes possibly related to pNET pathogenesis and progression, and hypermethylation of the promoters of *RASSF1*, *CDKN2A*, *TIMP3*, *MGMT*, *MLH1* and *IGF2* genes has been reported (Table 2). Ras association domain family 1 (*RASSF1*) is a tumor suppressor gene consisting of eight exons alternatively spliced to encode 8 protein isoforms, RASSF1A-H. RASSF1A is involved in microtubule stabilization, cell cycle regulation and induction of apoptosis [24], and the aberrant methylation of its promoter has been observed in 60-100% of pNETs [25–29]. Of interest, the transcription levels of *RASSF1A* are inversely correlated with the degree of gene methylation [30], and *RASSF1A* hypermethylation seems to predict pNET malignant features such as larger tumor diameter, nodal involvement and hepatic metastases [26, 28]. Cyclin-dependent kinase inhibitor 2A (*CDKN2A*) encodes for the tumor suppressor protein p16, which concurs to regulate cell cycle progression by inhibition of the G1/S transition. In a study of 48 well-differentiated pNETs, hypermethylation of *CDKN2A* was observed in 40% of tumors and was significantly associated with decreased patient survival and early tumor recurrence after surgery [26]. Of note, *CDKN2A* hypermethylation seems to be a hallmark of gastrinomas, since it occurs in 52-62% of gastrinomas but only in 17% of insulinomas [31–33]. Loss of p16 as result of gene promoter methylation is not associated with disease stage or prognosis, thus suggesting its early occurrence in gastrinoma pathogenesis [31, 32]. *In vitro*, reacquisition of p16 expression after treatment with the hypomethylating agent 5-aza-2′-deoxycytidine (decitabine) resulted in growth inhibition of pNET cells [34]. Methylation of the tumor suppressor tissue inhibitor of metalloproteinase-3 (*TIMP3*) has been found in 8/18 (44%) samples of pNETs, and has been correlated with loss or reduction of protein expression. *TIMP3*-negative tumors are at increased risk of lymph node and/or liver metastases, whereas they are apparently never associated with ectopic insulin production [35]. The suicide enzyme O^6^-methylguanine DNA methyltransferase (MGMT) repairs DNA by removing the O^6^-alkylguanine adducts, and its role in pNET chemoresistance to alkylating agents including temozolomide has been widely investigated, with controversial results [36–39]. Methylation in the promoter region of *MGMT* has been observed in up to 56% of pNETs, and only a partial concordance with protein expression has been demonstrated, thus suggesting the existence of various mechanisms of MGMT expression regulation in addition to transcriptional modulation [38]. Both MutL homolog 1 (*MLH1*) and Insulin-like growth factor 2 (*IGF2*) are frequently methylated in insulinomas, but not in other pNETs [40, 41]. Accordingly, in a recent study investigating the DNA methylation level of 807 cancer-related genes in insulinomas, gastrinomas and non-functioning pNETs, DNA methylation patterns were found to be specific for each tumor subtype [42]. An overview of recurring epigenomic difference between insulinomas and other pNETs is provided in Figure 2. Along with fundamental difference in the genetic background [3, 43], epigenetic changes peculiar of insulinomas might contribute to determine their unique clinical behavior.

**Table 2:** Incomplete list of epigenetic changes in sporadic NETs by primary site

NET primary site	Epigenetic alteration	Reference
Pancreas	Promoter hypermethylation: *RASSF1A* *CDKN2A* *TIMP3* *MGMT* *MLH1* *IGF2* *Axin-2, SFRPs* CIMP positivity Histone modifications: Upregulation of histone H3K9me2 miRNA upregulation: miR-103, −107, −23a, −26b, −192, −342 miR-144/451 miRNA downregulation: miR-155 IncRNA downregulation: *MEG3*	[27–31] [28, 33–35] [37] [40] [42] [43] [46] [31] [46] [51] [52] [51] [53]
Small bowel	Promoter hypermethylation: *RASSF1A* *TCEB3C* *THBS1, MGMT, p14, p16* *LAMA3, SERPINB5, RANK* Global hypomethylation Histone modifications: Upregulation of histone H1x Upregulation of H3K4diMe miRNA upregulation: miR-183, −488, - 19a+b miR-96, −182, −183, - 196a, 200a miRNA downregulation: miR-133a, −145, −146, −222, −10b miR-31, −129-5p, 133a, −215	[54] [58] [59] [5] [61, 63] [64] [65] [66] [67] [66] [67]
Lung	Promoter hypermethylation: *RASSF1A* *P15INK4b* Histone modifications: Downregulation of H4KM20 and H4KA16 miRNA upregulation: miR-129, −323-3p, −487b, −410, −369-3p, 376a miRNA downregulation: miR-203, −224, −155, −302, −34b, −181b, −193a, −5p, −34b	[69] [70] [73] [74] [74]

Hyperactivation of the Wnt/β-catenin signaling contributes to the pathogenesis and progression of pNETs. Although mutations of *β-catenin* or Wnt antagonists such as *APC* are rare, epigenetic silencing of Wnt inhibitors including *Axin-2*, secreted Frizzled-related proteins (*SFRPs*), Wnt inhibitory factor-1 (*WIF-1*) and DICKKOPFs (*DKKs*) has been reported in pNET cell lines. In particular, while silencing of *SFRPs* and *Axin-2* was related to the promoter methylation, downregulation of *WIF-1*, *DKK-1* and *DKK-3* was caused by repressive histone modifications leading to increased H3K9me2 presence at promoter level. Interestingly, treatment with decitabine was able to restore the expression of these genes, resulting in tumor suppressor functions both *in vitro* and *in vivo* [44].

Tumors that are characterized by frequent promoter methylation of tumor suppressor genes harbor the so called CpG island methylator phenotype (CIMP). CIMP positivity has been observed in 83% of pNETs *versus* 66% of extra-pancreatic NETs, and is apparently correlated with high proliferation index (Ki-67 > 10%) and poor prognosis, at least based on descriptive survival analyses [29]. In a study of 56 low-to-intermediate grade pNETs, methylation-sensitive multiple ligation-dependent probe amplification and long-interspersed nucleotide element-1 (LINE-1) analysis were used to assess tumor hypermethylation and hypomethylation status respectively. Futhermore, unsupervised hierarchical clustering allowed to separate a group of pNETs lacking both hyper- and hypomethylation features, a group characterized by moderate promoter hypermethylation as well as LINE-1 hypomethylation, and a highly hypermethylated group. The highly hypermethylated tumor phenotype predicts unfavorable disease progression, and is apparently associated with stage IV [29, 45].

Recently, a genome-wide DNA methylation study has been performed to assess the epigenetic consequences of DAXX/ATRX loss in pNETs. Alterations in the DNA methylation profile were more profound in DAXX-negative pNETs rather than in ATRX-negative or DAXX/ATRX-positive tumors, suggesting that the deficiency of DAXX, but not ATRX, drives genome-wide methylation changes leading to pNET formation [46]. DAXX and ATRX exert multiple functions including chromatin remodeling during heterochromatin assembly at repetitive guanine-rich regions, where they are required for incorporation of the histone variant H3.3 [47]. Mutations of *DAXX* or *ATRX* are mutually exclusive, and result in nuclear protein loss with consequent activation of the alternative lengthening of telomeres (ALT) pathway, a mechanism of telomerase-independent telomere maintenance. This eventually leads to chromosomal instability (CIN), tumor heterogeneity and metastases [48]. Histone modifications have been poorly studied in pNETs. However, the recurrent mutations of *MEN1* in sporadic pNETs suggest covalent histone changes as possible drivers of disease pathogenesis.

Studies of miRNAs in pNETs are scarse. In a series of pancreatic cancers and normal tissue, overexpression of miR-103 and miR-107 with concurrent miR-155 downregulation was distinctive of tumors, but did not separate pNETs from pancreatic acinar carcinomas. As compared with non-functioning pNETs, insulinomas were enriched in miR-204 [49]. In another study, a peculiar miRNA expression profile was shown to distinguish between insulinomas and normal pancreatic islets, and the miRNA cluster miR-144/451 was found to promote NET cell proliferation through suppression of the PTEN/AKT pathway [50]. Long non-coding RNAs (IncRNAs) have been implicated in pNET pathogenesis downstream of MEN1 biallelic inactivation. In particular, the IncRNA maternally expressed gene 3 (*MEG3*) is downregulated in pNETs, thus leading to the overexpression of its target protooncogene c-Met and consequently increased cell proliferation [51].

### Small bowel NETs

Similarly to pNETs, downregulation of RASSF1A as result of gene promoter methylation has been implicated in the progression of small bowel carcinoid tumors. In a study of 33 small bowel NETs and matched metastases, methylation levels of *RASSF1A* and *CTNNB1*, the gene encoding β-catenin, were significantly higher in metastatic tissue as compared with primaries. Of interest, both genes were unmethylated in an additional cohort of 6 nonmetastatic appendiceal carcinoids, thus suggesting that methylation of these genes is required for metastasis development [52]. More recently, increased methylation and consequent transcriptional repression of *RASSF1A*, but not *CTNNB1*, has been shown in metastases rather than small bowel primary NETs. Notably, low expression of RASSF1A significantly predicted poor survival and was associated with loss of chromosome 16q. *In vitro*, treatment with decitabine resulted in restored expression of RASSF1A in NET cell lines [53].

DNA methyltransferases (DNMTs) are the enzymes primarily responsible for DNA methylation. In particular, while DNMT1 maintains genome methylation during cellular replication, DNMT3a and DNMT3b act as *de novo* methyltransferases [54]. In a series of 63 foregut, midgut and hindgut GEP-NETs, DNMT1, 3a and 3b were expressed in 87%, 81% and 75% of samples respectively, and their expression was significantly higher in stage IV tumors. Of interest, DNMT3a and 3b were significantly down-regulated in midgut tumors relative to the foregut or hindgut NETs [55], thus suggesting that distinct epigenetic alterations characterize these diverse entities. Transcription Elongation Factor B Polypeptide 3C (*TCEB3C*) encodes for Elongin A3, which interacts with Elongin BC thus increasing the RNA polymerase II transcription elongation potential. *TCEB3C* is the only known imprinted gene on chromosome 18, and frequent loss of chromosome 18 has been reported in small bowel NETs. Epigenetic repression of *TCEB3C* has been recently observed in midgut carcinoids, and is apparently related to both DNA and histone methylation. Both decitabine, a demethylating agent, and the histone methyltransferase inhibitor 3-deazaneplanocin A and DNMT1 silencing *via* siRNA induced *TCEB3C* expression, thus leading to decreased carcinoid cell survival [56]. Using a candidate gene-driven approach, Chan *et al*. demonstrated that *p14*, *p16*, thrombospondin 1 (*THBS1*) and *MGMT* were selectively hypermethylated in midgut carcinoids when compared with the adjoining normal mucosa. CpG island methylation of *p16* also correlated with patient age and metastatic status [57]. CIMP positivity has been demonstrated in up to 29% of well-differentiated foregut and midgut NETs, and did not affect patient survival [29].

Although its importance is often underestimated as compared with the hypermethylation of the promoter of tumor-suppressor genes, global hypomethylation is a key epigenetic feature of human tumors. It refers to the methylation levels of DNA repeat elements such as short interspersed (Alu) and long interspersed nucleotide elements (LINE-1), which constitute approximately half of the genome, and is correlated with chromosomal instability and increased tumor mutation rate [58]. Contrasting results have been reported so far regarding the global methylation pattern of small bowel carcinoids. By LINE-1 and Alu methylation analyses, intestinal NETs were shown to be hypomethylated as compared with normal tissue, and this feature was more prevalent in ileal carcinoids rather than in other NETs, and was associated with lymph node metastases [59]. However, in a subsequent study, hypomethylation was more marked in pNETs, while NETs metastatic to lymph nodes were less frequently hypomethylated than nonmetastatic tumors [60]. More recently, Fotouhi *et al*. observed significantly lower methylation levels in small bowel carcinoids when compared to normal ileum, as well as in distant metastases rather than in primary tumors. Global hypomethylation was also associated with chromosome 18 loss [53]. In an attempt to define the epigenetic changes that take place during metastatic progression, the methylation profile of 10 small intestine NETs and 10 matched lymph node metastases has been recently compared. Overall, hypomethylation was more pronounced in metastatic tissue, and a large number of genes regulating cell growth, apoptosis, proliferation and metastasis formation were found to be differentially methylated between primaries and metastases. However, definite conclusions were hindered by the small sample size of the study [61].

In a large-scale multilocus analysis of DNA methylation patterns, two distinct groups of small intestine NETs were identified based on their DNA global methylation profile. Of interest, these two groups did not show any histological differences, including proliferation index, and were not associated with other known prognostic factors, thus suggesting possible pathogenetic differences lacking relevant consequences on tumor behavior [42]. Recently, Karpathakis *et al*. [5] have characterized 97 small bowel NETs by genetic, epigenetic and transcriptional profiling, and identified three subgroups of tumors by integrated molecular analysis. The largest group of NETs was defined by chromosome 18 loss of heterozygosity, and was associated with *CDKN1B* mutations and CIMP negativity. Patients of this subgroup had the most favorable progression-free survival (PFS) after resection and presented at an older age, thus suggesting a less aggressive tumor phenotype. On the other hand, a second subgroup was characterized by the absence of arm-level copy number variations (CNVs), a high level of CIMP and an intermediate PFS, while the third subgroup was defined by multiple CNVs, younger age at onset and dismal PFS. Overall, up to 85% of small bowel carcinoids harbored epimutations, and 21 epigenetically silenced genes were identified. Among those, there were genes located on chromosome 18 such as laminin alpha 3 (*LAMA3*), serpin peptidase inhibitor clade B member 5 (*SERPINB5*) and receptor activator of nuclear factor-κB (*RANK*). Because of the frequent LOH of chromosome 18 in small intestine NETs, epigenetic dysregulation of these genes has been proposed as the “second hit” capable to drive the tumorigenic process.

Studies on histone modifications in small bowel NETs are limited. Overexpression of histone variant H1x in gastrointestinal NETs seems to reflect its abundance in normal neuroendocrine cells, and might be useful for differential diagnosis in equivocal cases [62]. In 16 small intestine NETs, high levels of dimethylation of histone H3 at lysine 4 (H3K4diMe) have been observed, but further studies are needed to define the molecular implications of this finding in NET pathogenesis [63].

Small bowel NET progression is apparently characterized by a differential pattern of miRNA expression. In a study of 8 ileal carcinoid tumors, miR-183, −488, and 19a+b were up-regulated while miR-133a, −145, 146, −222 and −10b were down-regulated in metastatic tissue with respect to primary tumors [64]. In a subsequent study, 24 small intestinal NETs at different stages were profiled in their miRNA expression, and up-regulation of miR-96, −182, −183, −196a and 200a as well as down-regulation of miR-31, −129-5p, −133a and −215 were reported during tumor progression [65]. Up-regulation of miR-183 and down-regulation of miR-133a in both studies makes these miRNAs appealing targets for future investigations. Moreover, recent evidence suggests that miR-129-5p may have an anti-proliferative and anti-metastatic effect in midgut carcinoid tumors, and that its down-regulation during tumor progression might affect factors involved in RNA binding and nucleotide metabolism such as EGR1 and G3BP1 [66].

### Lung NETs

Chromatin remodeling and epigenetic dysregulation have been depicted as key events in the pathogenesis of pulmonary carcinoids. A recently published whole-genome/exome sequencing study of 69 lung NETs demonstrated mutually exclusive mutations of histone covalent modifiers in 40% of the samples. Among these genes, there were the menin-binding protein *PSIP1*, the histone methyltransferases *SETD1B*, *SETDB1* and *NSD1*, the histone demethylases *KDM4A*, *PHF8* and *JMJD1C*, as well as members of the Polycomb repressive histone H3K27 methyltransferase complexes *CBX6*, *EZH1* and *YY1*. Numerous mutations were also found in ATP-dependent chromatin remodeling SWI/SNF complex members including *ARID1*, *ARID2*, *SMARCA1*, *SMARCA2*, *SMARCA4*, *SMARCC2*, *SMARCB1* and *BCL11A*. Given the almost universal absence of other cancer-related mutations, alteration of chromatin-remodeling pathways is apparently sufficient to drive early steps of lung NET tumorigenesis [2].

As previously reported for pNETs and small bowel carcinoids, *RASSF1* promoter hypermethylation is a frequent event in low-to intermediate grade lung NETs. Of interest, the degree of promoter methylation was associated with tumor grade, while there was a non-linear correlation between levels of methylation and *RASSF1* mRNA or protein content, thus suggesting that other transcriptional or post-transcriptional events may concur in its regulation [67]. In a series of 5 low-grade and 15 high-grade lung NETs, aberrant methylation at the 5′-region of the *p15INK4b* gene was observed in 15% of tumors, but not in normal bronchial tissue. *p15INK4b* encodes for a cyclin-dependent kinase inhibitor, and has recently emerged as a possible regulator of tumorigenesis *via* cell cycle dysregulation [68]. The protein arginine methyltransferase-5 (PRMT5) is a chromatin-modifying enzyme, and is overexpressed in bronchial NETs. In particular, nuclear overexpression of PRMT5 was negatively associated with tumor grade, thus reflecting potential differences in the epigenetic control of oncogenesis of low- and high-grade lung NETs [69]. Consistently with this hypothesis, expression levels of the histone methyltransferase enhancer of zeste homolog 2 (*EZH2*) have been shown to inversely correlate with tumor grade in lung NETs [70].

Comprehensive analyses of histone modifications in bronchial carcinoids are lacking. In a study of 32 lung NETs, histone H4 acetylation at lysine 16 (H4KA16) and trimethylation at lysine 20 (H4KM20) were studied. As in other cancers, a progressive loss of H4KM20 and H4KA16 was observed during progression from normal bronchiolar epithelium to low grade tumors and then to high-grade carcinomas. The biological implications underlying this finding need to be clarified [71].

Several studies have recently profiled the expression of miRNAs in pulmonary carcinoids, reporting differences between normal lung tissue and tumor, low- and high-grade bronchial NETs as well as localized and metastatic disease [72–75]. Among recurrently deregulated miRNAs in pulmonary carcinoids, miR-21, miR-155 and miR-129-5p were already identified as possible regulators of pNET or midgut NET pathogenesis, thus suggesting their pivotal role in NET oncogenesis and progression.

## CLINICAL ONCOEPIGENOMICS OF NETS IN THE PRECISION MEDICINE ERA

Precision medicine refers to individualized prevention and treatment of diseases based on their underlying molecular causes, and carries the promise of coupling established clinical-pathological parameters with state-of-the-art molecular profiling to create diagnostic, prognostic and therapeutic strategies tailored to each patient's requirements. Data from the Human Genome Project and the global diffusion of next-generation sequencing technologies have dramatically advanced the practice of personalized medicine, and many cancers are currently treated using agents targeting underlying aberrant genomic changes [76].

The genomic landscape of NETs has been decoded only very recently, and the clinical relevance of peculiar mutational profiles is still under investigation. However, no genetic signature can be used for treatment tailoring in NET patients at present, even when driver mutations affect the signaling pathways targeted by currently approved drugs. While genetic features of different site NETs are extremely heterogeneous, a limited number of pathways seem to have a role in their pathogenesis, as demonstrated by the almost universal efficacy of somatostatin analogs and mTOR inhibition in these tumors [77, 78]. Since disruption of the epigenetic machinery is common to all NETs, a possible molecular reunification of such a diverse disease can be envisaged, thus explaining their similar response to several therapeutic agents. Although the NET epigenome characterization is still in its infancy, epigenetic profiles have already demonstrated clinical utility for the diagnosis and prognosis of NETs, and might pave the way to new personalized therapeutic strategies.

### Epigenetic profiles in NET diagnosis and prognosis

A whole-genome DNA methylation analysis of 12 normal lung tissue samples and 124 lung tumors has recently identified five DNA methylation subgroups. Of note, one epitype was distinctly associated with neuroendocrine differentiation [79]. Similarly, small bowel carcinoids and distinct pNET subtypes have been shown to harbor peculiar DNA methylation patterns [42]. Discrimination of the various subtypes of pulmonary NETs *via* miRNA profiling has been demonstrated to be feasible [80]. Recently, an integrated multi-omics data analysis has assessed the transcriptomic (mRNA and miRNA), mutatomic (selected mutations) and metabolomic profile of pNETs, identifying three distinctive molecular subtypes. While the islet/insulinoma tumor (IT) subtype was characterized by low grade and low metastatic potential, the metastasis-like primary (MLP) subtype displayed high proliferative activity, a clinically aggressive behavior and a gene expression profile consistent with epithelial-to-mesenchymal transition (EMT). On the other hand, the MEN1-like/intermediate subtype was enriched in *MEN1* mutations, and showed moderate metastatic potential. Although validation studies are needed, this seminal molecular classification of pNETs shows promising clinical applicability, potentially leading to a better therapeutic planning [81]. Observations from our group are also in line with this classification. In fact, we have previously reported that osteotropic NETs overactivate EMT, while being characterized by very dismal prognosis [82]. Given the apparent pivotal role of CXCR4 in inducing EMT and metastases in NETs (manuscript submitted), and the robust epigenetic regulation of the CXCL12/CXCR4 axis in cancer [83], future work should determine if complex NET epigenetic profiles and resulting patterns of biologic malignancy as well as clinical outcomes may be recapitulated, at least in part, by CXCR4 expression.

A multianalyte whole blood RNA multigene signature has been developed to predict NET activity. Interestingly, among the omic clusters capable of differentiating disease activity (stable *versus* progressive disease), there was the epigenome [84]. Future studies should assess if the epigenomic profiling *per se* may serve as a predictive biomarker for treatment tailoring in NET patients. In this context, the epigenetic silencing of *MGMT* has been described as predictive of response to alkylating agents in pNETs [38, 39], but prospective validation is still lacking. The prospective randomized trial NCT01824875, investigating temozolomide alone or in combination with capecitabine in pNET patients, incorporates *MGMT* promoter methylation testing, and may therefore provide further insight on this topic.

Epigenetic clustering has shown prognostic relevance in GEP-NETs, while studies in bronchial carcinoids are lacking. In low-to-intermediate pNETs, the combination of DNA global hypomethylation and cluster gene hypermethylation significantly predicted patient outcomes in multivariable analysis [45]. In addition, high expression of miR-196a is apparently associated with decreased overall survival (OS) and disease-free survival (DFS) in pNET patients [85]. As described above, the CIMP status has demonstrated prognostic relevance in small bowel NETs, with a substantial impact on patient PFS [5].

### Epigenetic therapy of NETs

Since alterations of the chromatin remodeling machinery seem to be major drivers of NET development, epigenetic agents such as DNMT antagonists or HDAC inhibitors may be effective for patients with NETs. Moreover, in contrast to DNA mutations, DNA methylation and histone modifications are reversible and seem to be a feature defining cancer stem cells [86], thus representing an appealing therapeutic target in cancer. An overview of the epigenetic agents currently approved by FDA for cancer patients, their mechanism of action and clinical indications is provided in Table 3.

**Table 3:** Approved epigenetic agents in the treatment of cancer in Europe and North America

Drug	Mechanism of action	Approved indications	First FDA approval
Azacitidine	Inhibition of DNA methyltransferases	Myelodysplastic syndromes (FDA, EMA) Acute myeloid leukemia (EMA) Chronic myelomonocytic leukemia (EMA)	2004
Decitabine	Inhibition of DNA methyltransferases	Myelodysplastic syndromes (FDA) Acute myeloid leukemia (EMA)	2006
Vorinostat	Inhibition of histone deacetylases (class I and II)	Cutaneous T-cell lymphoma (FDA, EMA)	2006
Romidepsin	Inhibition of histone deacetylases (1,2,4,6)	Cutaneous T-cell lymphoma (FDA)	2009
Belinostat	Inhibition of histone deacetylases (class I, II and IV)	Peripheral T-cell lymphoma (FDA)	2014
Panobinostat	Inhibition of histone deacetylases (class I and II)	Multiple myeloma (FDA, EMA)	2015

The efficacy of DNMT inhibitors in NETs has been tested only *in vitro* thus far. Azacytidine caused a dose-dependent reduction of the proliferation of CNDT2.5, H727 and BON1 cell lines [87]. Similarly, the demethylating agent decitabine showed antiproliferative effects on QGP1 pNET cells, possibly as result of the restoration of multiple genes silenced by pathogenetic *de novo* methylation. In particular, after treatment with the drug, tumor cells expressed a differential pattern of genes involved in proliferation, apoptosis and metastases [34]. Decitabine was also able to restore the expression of *RASSF1A* in bronchial NET cell lines [52].

HDAC inhibitors such as trichostatin A, sodium butyrate and entinostat have been tested on pNET cell lines, and caused a dose-dependent inhibition of proliferation, cell cycle arrest and apoptosis. No synergistic effects were noted after combination with somatostatin or octreotide [88]. The antiproliferative effects of valproic acid (VPA) as a class I and IIa HDAC inhibitor have been extensively investigated in NETs. The drug is able to induce a dose-dependent growth inhibition of NET cells of pancreatic and intestinal origin *in vitro* through activation of both intrinsic and extrinsic apoptosis. Of note, expression of up to 20% of protein-coding genes was significantly modified by VPA in NET cell lines, leading to major alterations in key regulatory pathways including the signaling of p53, TGFβ1 and MYC [89]. VPA has also been demonstrated to up-regulate Notch1 and somatostatin receptor subtype 2 (SSTR2). While activation of Notch1 is able *per se* to suppress NET tumor growth and is associated with reduction of NET tumor markers [90, 91], SSTR2 up-regulation has been used to synergistically increase the cytotoxicity of a SSTR-targeting camptothecin-somatostatin conjugate [92]. Concurrent activation of the Notch1 pathway and inhibition of the GSK3β signaling by combination of VPA and lithium has been reported to suppress NET cell proliferation, while decreasing CgA production [93].

Early evidence showed that VPA exerts antisecretory effects in NETs, significantly decreasing the plasma concentration of somatostatin in a single patient with somatostatinoma [94]. In a pilot phase II study of eight patients with low-grade pNETs or midgut carcinoids, VPA at 500 mg daily induced an unconfirmed partial response and disease stabilization in one and four subjects respectively. Of note, Notch1 upregulation following treatment was apparently associated with better outcomes [95]. The HDAC inhibitor depsipeptide has been investigated in a phase II study of 15 patients with metastatic NETs. The trial has been prematurely discontinued due to an unexpected rate of severe cardiac toxicities, including a fatal ventricular arrhythmia [96]. In a single arm phase II study of 15 patients with GEP-NETs, panobinostat was associated with a 90% rate of disease stabilization and a median PFS of 11.8 months. The trial was stopped at interim analysis due to lack of objective responses [97]. However, it should be noted that response rate is currently not considered an optimal endpoint for NET clinical trials [98]. The DNA methylation and deacetylation inhibitor RRx-001 has recently demonstrated to inhibit hormone release in a single patient with carcinoid syndrome, leading to rapid symptom reversal and improved quality of life [99]. However, future studies are needed to confirm this observation.

Modulators of H3K4me3 demethylases of the KDM5 family are currently being developed, and have already shown significant antitumor activity in murine models of MEN1 knock-out pNETs [20]. Inhibition of KDM5 proteins could be an attractive future strategy in NET patients, and MEN1 deficiency might be envisaged as a potential predictor of response. Although preclinical evidence of efficacy for epigenetic agents in NETs is promising, no significant benefit has been demonstrated so far by clinical trials. However, as a possible explanation for this lack of efficacy, it should be noted that these studies were carried out using HDACi, while the preclinical investigations of epigenetic agents in NETs have prevalently used demethylating agents. The only epigenetic modulator still under clinical investigation is belinostat, which is currently being trialled in combination with cisplatin and etoposide, in patients with advanced neuroendocrine carcinomas (https://clinicaltrials.gov/ct2/show/study/NCT00926640?term = nct00926640&rank = 1).

## CONCLUSIONS AND FUTURE PERSPECTIVES

Since commonly mutated oncogenes play little or no pathogenetic role in NETs, epigenetic alterations are likely to be major determinants of NET tumorigenesis. However, whether the observed modifications of DNA packaging are driver or passenger events, and their position and role in the evolutionary tree of NETs still remains to be clarified. Recently, several studies have compared the genomic make-up of NET cell lines to the genetic signature of primary NETs, demonstrating striking differences in the rates and patterns of mutations [100, 101]. Future work is needed to assess the degree of overlap between the epigenetic landscape of NET cell lines and correspondent patient tumors. In the meanwhile, the results from experiments with NET cell lines should be interpreted with caution, and direct clinical extrapolation should be avoided.

Although profiles of the epigenome of NETs have been already proposed, results need to be validated in larger studies. The identification of molecularly different NET subtypes might have a dramatic impact on clinical practice, potentially leading to new diagnostic classification, prognostic stratification and innovative clinical trials. While precision medicine has thus far led to the “explosion” of each form of cancer in multiple distinct diseases, the common epigenetic changes seen in a wide spectrum of NETs have the potential of re-unifying such diverse clinical entities. Future investigations should verify if the epigenetic fingerprinting of NETs might provide better clinical classification/prognostic stratification than site of disease or grade. No biomarker-driven clinical trials of epidrugs have been carried out in NETs so far, thus possibly explaining the observed lack of significant efficacy with these agents. Similarly to studies of therapeutics targeting discrete genomic mutations, innovative basket trials testing epigenetic drugs should ideally include molecularly defined subpopulations of NET patients, thus paving the way to studies dedicated to emerging homogeneous epitypes of NET patients.

Since NETs originate from neuroendocrine cells, studies of epigenetic profiling should be carried out with appropriate control tissue. This might be a potential drawback for a precise characterization of small bowel or bronchial carcinoids, but innovative methods for single-cell genome-wide epigenomic analyses are now on the horizon [102]. Recent evidence [103, 104] suggests that epidrugs stimulate the expression of benign retroviruses inserted in the genome of all human cells, including tumor cells. As result, a “viral mimicry” phenomenon and its beneficial effects on tumor immunogenicity have been described, thus providing a rationale for possible combination studies in NETs with epidrugs and immune checkpoint inhibitors such as nivolumab or ipilimumab [105]. Impressive progress has been made in our understanding of NET pathogenesis in recent years, and the recognition of pivotal pathways of tumorigenesis has led to the approval of new targeted therapies for NET patients. Outstanding bench to bedside and back work is now again needed to further move from NET treatment to NET cure.
